# Process evaluation of a point-of-care cluster randomised trial using a computer-delivered intervention to reduce antibiotic prescribing in primary care

**DOI:** 10.1186/s12913-014-0594-1

**Published:** 2014-12-03

**Authors:** Lisa McDermott, Lucy Yardley, Paul Little, Tjeerd van Staa, Alex Dregan, Gerard McCann, Mark Ashworth, Martin Gulliford, The eCRT research team

**Affiliations:** Department of Primary Care and Public Health Sciences, King’s College London, Capital House, 42 Weston Street, London, UK; Department of Psychology, University of Southampton, Shakleton Building, Highfield, Southampton, UK; Aldermoor Health Centre, School of Primary Care and Population Sciences, University of Southampton, Aldermoor Close, Southampton, UK; The Clinical Practice Research Datalink Group, The Medicines and Healthcare products Regulatory Agency, 5th Floor, 151 Buckingham Palace Road, London, Victoria UK

**Keywords:** Cluster trial, Pragmatic trial, Point of care, Antibiotic utilisation, Primary care, Implementation science

## Abstract

**Background:**

The study aimed to conduct a process evaluation for a cluster randomised trial of a computer-delivered, point-of-care intervention to reduce antibiotic prescribing in primary care. The study aimed to evaluate both the intervention and implementation of the trial.

**Methods:**

The intervention comprised a set of electronic educational and decision support tools that were remotely installed and activated during consultations with patients with acute respiratory infections over a 12 month intervention period. A mixed method evaluation was conducted with 103 general practitioners (GPs) who participated in the trial. Semi-structured telephone interviews were conducted with 20 GPs who had been in the intervention group of the trial and 4 members of the implementation staff. Questionnaires, consisting of both intervention evaluation and theory-based measures, were self-administered to 83 GPs (56 control group and 27 intervention group).

**Results:**

Interviews suggested that a key factor influencing GPs’ use of the intervention appeared to be their awareness of the implementation of the system into their practice. GPs who were aware of the implementation of the intervention reported feeling confident in using it if they chose to and understood the purpose of the intervention screens. However, GPs who were unaware that the intervention would be appearing often reported feeling confused when they saw the messages appear on the screen and not fully understanding what they were for or how they could be used. Intervention evaluation questionnaires indicated that GPs were satisfied with the usability of the prompts, and theory-based measures revealed that intervention group GPs reported higher levels of self-efficacy in managing RTI patients according to recommended guidelines compared to GPs in the control group.

**Conclusions:**

Remote installation of a computer-delivered intervention for use at the point-of-care was feasible and acceptable. Additional measures to promote awareness of the intervention may be required to promote health care professionals’ utilisation of the intervention and these might sometimes compromise the pragmatic intention of a trial.

**Trial registration:**

ISRCTN47558792 (registered on 17 March 2010).

## Background

Non-adherence to clinical guidelines by health professionals has frequently been reported across a range of conditions [1]. In particular, non-adherence to recommended guidelines has consistently been reported in primary care [2,3]. Failing to adhere to guidelines in primary care can lead to a number of complications including worsening of a patient’s medical condition or an increase in the risk of related conditions. As practitioner non-adherence to clinical recommendations can lead to potentially serious health outcomes for patients, a number of implementation techniques to encourage adherence to guidelines have been used. Inventions have included educational programmes and materials, patient materials, reminders and computer delivered systems [4,5]. Specifically, ‘point of care’ interventions which appear during a consultation or at the point of practitioner decision making appear to be more effective [6,7] and can both improve patient care and change healthcare professionals’ behaviour [8,9].

Point of care interventions are increasingly being delivered electronically by computer, drawing on the capability of systems to present reminders and advice in a ‘real time’ context within the consultation [10]. There is increasing evidence to support the value of computer delivered interventions to improve GPs’ adherence to clinical guidelines and patient-related outcomes [11,12]. In particular, computer-based interventions which use reminders and automatic prompts have been found to be most successful [13]. However, success rates of computer-delivered interventions vary considerably, with increased adherence to clinical guidelines ranging from 3% [14] to 42% [12] changes in prescribing. A large scale review, involving 100 trials, concluded that computer-based decision support systems can improve practitioner performance but that the effects of such interventions remain both understudied and inconsistent [13,15].

We previously developed and reported a computer delivered ‘point of care’ intervention to promote appropriate antibiotic prescribing for RTI (Respiratory Tract Infection) in primary care [16]. The intervention content was informed by the UK National Institute for Health and Care Excellence (NICE) [17] recommendations which advice that antibiotics should not be prescribed to most patients with a RTI, and that a prescription of antibiotics should only be issued to patients with specific additional underlying medical conditions and high risk groups. However, GPs often prescribe antibiotics unnecessarily, which can contribute to the spread of resistant bacteria and result in antibiotics which are ineffective [18-20] highlighting the need for intervention.

The intervention we developed aimed to increase GP adherence to guidelines for antibiotic prescribing in primary care. The intervention was informed by behaviour change theory [5,21,22] and was developed using feedback from qualitative interviews with GPs. A combination of interview techniques were used including ‘think aloud’ interviews during which a GP would respond to the use of a pilot version of the intervention. The intervention was then implemented in a cluster randomised trial with 104 GP practices randomised, (the protocol has been reported in Gulliford et al. [23]). The intervention was installed remotely through a system known as DXS Point-of-Care. The intervention was activated during consultations with patients who were presenting with a RTI. The electronic support tools included a summary of antibiotic prescribing recommendations, a printable patient information sheet, a summary of research evidence concerning no antibiotic or delayed antibiotic prescribing strategies, information on the definite indications for antibiotic prescription, as well as information and evidence on the risks from non-prescribing. The decision support tools included separate modules for sore throat, cough and bronchitis, otitis media, rhino-sinusitis, and common colds. The GP could choose to click on any of the additional pages, or ignore the message and continue with the consultation. Throughout this article we will refer the pages of the computer delivered intervention we previously developed as ‘prompts’.

The aim of this study was to conduct a process evaluation for the trial of a computer delivered intervention to reduce antibiotic prescribing in primary care [23]. This was a new study which did not include data from the cluster trial. The study aimed to evaluate the feasibility and acceptability of the intervention in clinical practice and evaluate the implementation of the intervention into a practice setting including factors affecting uptake and effectiveness.

## Methods

### Design of study

A mixed methods design was employed including an interview study and a questionnaire study. The study was conducted primarily with general practitioners who had participated in the trial of a computer-delivered intervention to reduce antibiotic prescribing for RTIs in primary care [23]. Staff involved with intervention implementation were also included.

### Participants

GPs from each of the 100 practices who had taken part in the trial were invited to participate (as demonstrated in Table 1). In total, 103 general practitioners and four members of staff involved in the implementation of the intervention took part in the evaluation. The interview study consisted of 24 participants (20 GPs and 4 members of the trial implementation staff). The interviews were only conducted with GPs who had been part of the intervention group and not the control group (as all questions related to the intervention). The questionnaire study consisted of 83 GPs (56 control group and 27 from the intervention group). The research was conducted at the end of the trial period, which lasted for one year in each practice. For the interview part of the study, all GPs were from intervention group practices as the questions related to use of the intervention. The implementation staff who took part in interviews included individuals from 3 organisations who had been involved in recruitment, software implementation and practice communication during the trial. For the questionnaire study, GPs from both the intervention and control groups were recruited to compare views towards antibiotic prescribing following the trial; the intervention group also took part in an additional questionnaire measure relating to the prompts.

**Table 1:** **Procedure used to recruit GPs**

**Timeframe**	**Intervention Group GP practices**	**Control Group GP practices**
During 1 year trial	Electronic reminder prompts appear during consultations for RTIs	No change to usual practice
3 weeks before trial end date	Receive invitation to take part in interview and questionnaire	Receive invitation to take part in questionnaire
2 weeks before trial end date	Receive reminder invitation to take part in interview and questionnaire	Receive invitation to take part in questionnaire
1 week before trial end date	Receive final reminder invitation to take part in interview and questionnaire	Receive final invitation to take part in questionnaire

As this evaluation related to a cluster randomised trial, demographics were collected at practice level. GPs from practices across the country took part in the questionnaire, these included: Surrey, London, Oxford, Devon, Birmingham, and Warwickshire. Surgery size varied across practices with the number of full time or equivalent GPs ranging from 1 to 12, and the number of patients registered with each full time equivalent GP ranging from 598 to 1194. The index of multiple deprivation score (IMD) also varied greatly and ranged from 7 to 47, with higher scores indicating greater deprivation.

### Procedure and materials

The study obtained Ethical approval from the South West London Research and Ethics Committee, Ref: 09/H0806/81 and NHS Research Governance approval as a voluntary component of the trial participation. Table 1 demonstrates the procedure used to recruit GPs for the study. The trial had been running for approximately one year prior to the evaluation being conducted. Invitations were sent via email and post, consecutively, and both electronic and paper copies of questionnaires were provided to GPs for self-completion. Invitations were sent to the individual at each practice who had consented to their surgery taking part in the trial. Practices contacted the researcher (LM) directly by email or telephone (contact details were provided in the invitation letters) to arrange a convenient interview time and/or ask questions regarding the nature of the interviews or questionnaires. Interviews were conducted by telephone and lasted for approximately 30 minutes. All interviews were recorded and transcribed verbatim prior to analysis.

The interviews were conducted by a member of the study team who was not involved in the trial management and at the time of the study was based in a separate institution (University of Southampton whilst the trial management took place at King’s College London. It was made clear to GPs at the time of the interview the interviewer was not involved in trial management of the study).

The interview and questionnaire development were guided by the key criteria suggested by Linnan and Steckler [24] for the process evaluation of public health interventions and research. These components were evaluated across the questionnaires and interviews (Table 2). The GP interview guide used a series of open-ended questions in order to facilitate in-depth discussion of the prompts and trial. The interview was designed to discuss the use of prompts within a clinical setting, problems with the prompts or implementation, improvements which could be made, and to highlight any issues with the intervention which may not have previously been considered. A semi-structured interview guide was also constructed for members of staff who had been involved in the implementation of prompts. The questions were designed to explore factors which may have influenced the implementation of the intervention/prompts and allow the participant to describe their experiences. Interview guides are presented in Tables 3 and 4.

**Table 2:** **Process evaluation components and method used**

**Evaluation component**	**Evaluation method**
Context	Evaluation questionnaire/Interview
Reach	Interview
Dose delivered	Interview
Dose received	Evaluation questionnaire/Interview
Fidelity	Evaluation questionnaire/Interview
Theoretical constructs	Outcome Expectancies and Self-Efficacy Questionnaires

**Table 3:** **Semi-structured interview schedule for GPs**

**Question number**	**Question**
1	How did you feel about the prompts appearing?
2	How do you think patients felt about the prompts?
3	How much did you discuss the use of prompts with colleagues? (What did they think?).
4	Can you tell me about any situation where you successfully used the prompts?
5	Can you tell about any situation where you chose not to use the prompts.
6	Can you give me an example of a situation where you used the prompts but experienced a problem or difficulty?
7	How do you think the prompts could be improved or made easier to use?
8	Can you give me any examples of features of the prompts that you did not like?
9	How do you think the prompts impacted practice? (Any impacts on practice positive or negative. i.e. did they think the prompts 'worked'?)

**Table 4:** **Interview schedule for implementation staff**

**Question number**	**Question**
1	Could you briefly outline your role in the implementation of the RTI prompts used in this trial?
2	What do you think were the main challenges in setting up the trial?
3	How do you feel these may have impacted the trial overall?
4	How do you think the recruitment methods used to allocate practices may have influenced the trial?
5	How do you think the level of communication between the team and practices may have influenced the overall implementation of the trial?
6	Overall what do you think the main difficulties are in implementing an intervention of this nature?
7	What do you think could be done in the future to improve the implementation of interventions like these?
8	Finally, do you have any further comments you’d like to add based on your experience of this intervention?

The intervention group received all 3 questionnaire measures described below. The Outcome Expectancies and Self-Efficacy questionnaires assessed the two theoretical constructs proposed by Bandura’s social cognitive theory [21] to be the key predictors of behaviour. The control group received only the outcome expectancies and self-efficacy questionnaires (as they had not seen the intervention therefore could not evaluate it). Both online and paper versions of the questionnaires were made available for GPs to choose from.

The outcome expectancies questionnaire was designed to measure GPs’ outcome expectancies relating to managing a patient with a RTI without prescribing antibiotics (in accordance with the NICE, 2008 guidelines [17]). The questionnaire aimed to capture the outcomes which GPs expected to result from following the NICE guidelines and not prescribing antibiotics in the majority of patients with a RTI. The outcomes presented in each item were devised by identifying issues which had been highlighted in previous research as influencing GP’s decision to prescribe antibiotics to patients with a RTI. Each item asked the GP to rate their opinion on a five point scale from agree strongly to disagree strongly, in response to the statement “If I treat a patient with an RTI without prescribing antibiotics....” (which was followed by a likely outcome such as ‘The patient will be dissatisfied with the outcome’).

The self-efficacy questionnaire was designed to measure GPs’ self-efficacy in relation to managing a patient with a RTI without prescribing antibiotics (in accordance with the NICE, 2008 guidelines). The situations presented in each item were devised by identifying issues which had been highlighted in previous research as influencing GPs’ decision to prescribe antibiotics to patients with a RTI [5,16,18]. Each item asked the GP to rate their opinion on a ten point scale from ‘Cannot do’ to ‘Highly certain can do’, in response to the question:” How certain are you that you could treat a patient with an RTI without an immediate prescription of antibiotics?” (which was followed by a possible situation such as ‘When the patient DOES want antibiotics’).

The intervention evaluation questionnaire aimed to evaluate the intervention as part of a wider process evaluation in conjunction with the qualitative interviews. The components aimed to evaluate perceptions of the software, usefulness of each prompt type, use during a consultation, agreement with guidelines and communication within practices. The questions within the measure were divided as follows-

Section 1: Software: This section aimed to evaluate the functionality of the prompts which had been delivered during the intervention. The questions required the GP to rate their level of agreement with statements relating to the way in which prompts could be accessed, read and used during a consultation.

Section 2: Prompt type: This section aimed to assess how useful the prompts were for each condition in practice. The questions required the GP to rate their level of agreement with the statement that a prompt was useful in supporting practice (this was conducted for each type of prompt).

Section 3: Consultation: This section aimed to assess the ease with which the prompts could be used during a consultation.

Section 4: Additional issues: This section combined two topics: agreement with guidelines and communication.

Agreement with guidelines: These questions aimed to assess the degree to which GPs were familiar with and agreed with NICE guidelines [17] for the non-prescription of antibiotics in patients with RTI.

Communication: These questions aimed to assess the quality of communication relating to the prompts and implementation with GP practices.

### Analysis

For the interview data, inductive thematic analysis [25] was conducted on all transcripts to identify participants’ experiences of using the prompts and experiences of the study implementation. Analysis began after the first interview had been conducted and continued throughout data collection for all interviews conducted. In accordance with the Braun and Clarke [25] technique for thematic analysis the following methods took place:

1. Familiarization with data: Interview transcripts were read in detail and re-read. The coder took notes which may assist in the understanding of any aspects of the interviews.
2. Generating initial codes: Commonly occurring patterns and prominent themes were identified in the data and labelled with codes. As inductive thematic analysis was being conducted, the codes related directly to meaning presented in the text and did not attempt to present assumptions or theoretical concepts to the text sections being coded.
3. Searching for themes: Codes were grouped into potential themes which could best explain the codes which had emerged from the data.
4. Reviewing themes: A continuing process of revision occurred. This involved themes being linked, grouped, moved, re-labelled, added and removed to produce a set of themes which adequately fit and thoroughly explained the data.
5. Defining and naming themes: Over-aching themes were refined and defined in as much detail as possible to reflect exactly what was occurring within the text. A coding manual was used to assist with this process, which consisted of a full and thorough definition of each theme, an example of each instance of this theme within the transcripts, and supporting quotes.

Following this, inter-rater agreement was then reached on all themes following detailed discussion of every example and coded extract of transcript. This process was conducted by two experienced qualitative researchers (Authors LM and LY).

For the questionnaire, the two theory based measures (outcome expectancies and self-efficacy) were assessed for reliability using Cronbach’s alpha on both scales. The measures and items were compared for group differences using Kruskal-Wallis comparisons as the data were non-Normal. The intervention evaluation questionnaire was assessed for responses to each section and percentage scores for each question are described.

## Results

### Interview study

Four themes (Table 5) emerged from the interviews relating to GPs’ experience of using the prompts in practice and the study implementation. Themes were noted as being common across all interviews and did not differ across practice characteristics. Thematic saturation of themes was reached relatively early in the study after approximately half (10) of the sample had been interviewed. No new themes emerged in the second half of interviews, therefore the four overarching themes were considered to be highly relevant to the GPs experience of the intervention. Interviews from staff involved in the implementation of the study identified 2 key sub-themes (Table 5) relating to the trial implementation.

**Table 5:** **Themes and sub-themes identified in interviews**

**Interview group**	**Themes**	**Subthemes**
*GPs*	**Awareness of implementation**	-Aware of implementation and confident to use prompts
		-Unaware of implementation and confusion as to prompts’ purpose
	**Usefulness of prompts**	-Useful for inexperienced practitioners
		-Support for decision
		-A reminder/reference tool
		-Can help reduce prescribing
		-Not needed as guidelines already followed
	**Positive impact on patients**	-Assistance in persuading patients
		-Acceptable to patients
		-Patient information sheet very useful feature.
	**Usability issues**	-Easy to use
		-Easy to control
		-Limited time to read and use
		-Only English language available
		-Additional features
		-Simplify further
		-Increase visibility of prompts
*Implementation staff*	**Communication difficulties within practices**	-Delays due to practice staff unawareness of study.
		-Improvements to staff awareness needed.

GPs=general practitioners.

Description of GP themes (Over-arching theme is described followed by sub-theme description and example):

#### Awareness of implementation

The GPs’ level of awareness regarding the implementation of the prompts onto their system often influenced their willingness to use them or even whether they noticed them. Two distinct sub-themes were identified within this theme, relating to whether or not GPs were aware of the prompts. GPs who were aware of the prompts generally reported feeling confident in using them whereas GPs who were not aware of the prompts generally appeared confused as to their purpose. Therefore being aware of the prompts appeared to be related to a positive view towards usage. Detail of each sub-theme is explained below:

- Aware of implementation and confident to use prompts

GPs who reported being aware of the implementation of the prompts either before or during the study reported being confident to use the prompts if they chose or wished to. Confidence in using the prompts was reported as being a result of the fact that GPs who were aware of the study understood the prompts’ purpose, were expecting them, and considered the source of information within the prompts as plausible and trustworthy. Awareness of the study was reported as occurring via a number of methods which included; official emails detailing the implementation sent to all staff members; information presented during a staff meeting; informal discussions with colleagues; or informal discussions with a practice manager or practice research co-ordinator.

*"We talked about it in practice so I was expecting it….I thought it was a very useful aid for me" (P08)*

- Unaware of implementation and confusion as to purpose of prompts

GPs who reported being unaware of the implementation of the prompts often reported being confused about their purpose. GPs who saw the prompts appear but had not been formally made aware of the study or the appearance of prompts often reported being less likely to use or look at them for a number of reasons which included: a lack of understanding of the prompts’ function; uncertainty about the source of information; uncertainty about whether the information could be trusted; uncertainty about whether the prompts were an advertisement (which are often shown in the same screen location). GPs who were unaware of the implementation also appeared to be less likely to have noticed the prompts. However, during the interviews once the purpose of the prompts had been made clear, some GPs reported that they would have been happy to use and try the prompts if they had been aware of this information sooner.

*"I don't think anyone actually pointed it out to me…..I might have just though 'Oh is that some sort of advertisement'…I probably would have used it, but definitely I would you know" (P05)*

#### Usefulness of prompts

The prompts were often discussed in relation to their use as a tool in practice, either during a consultation or during the GPs’ own time.

- Useful for inexperienced practitioners

GPs often reported the prompts as being particularly useful for a group of staff which they described as ‘inexperienced practitioners’. GPs described inexperienced staff members as including newly qualified GPs, student doctors, locums, and nurse practitioners. The GPs interviewed in this sample reported that these ‘inexperienced staff’ may benefit from the prompts as they would be less aware of the guidelines in general, the evidence and the recommendations not to prescribe antibiotics. (However, transcripts did not provide details of the level of experience obtained by GPs within this sample).

*"New colleagues or new prescribers might be needing to look at it more" (P01)*

- Support for decision

The prompts were reported as providing GPs with support in prescribing decisions they had already made prior to reading the material or looking at the screen. This issue was reported as being closely related to providing the GP with confidence in their decision and presentation of advice not to prescribe antibiotics. GPs were happy and willing to engage with and use the prompts if they perceived them as a tool which could support their own decision to either not prescribe antibiotics or issue a delayed prescription.

*"First of all they give confidence to the doctor, that there is some evidence behind the decision" (P08)*

- A reminder/reference tool

The prompts were often described by GPs as a reminder or reference tool. GPs reported using the prompts as a reminder for the guidelines and in particular as a facility to obtain and read references for the evidence which supports the guidelines. The use of the prompts as a reminder and reference tool was often reported as being used outside of the consultation time when a GP would have more time to read through these details if required.

*"I think that it is a useful reminder, it doesn’t take long before having seen them and you've refreshed your memory of the NICE guidelines" (P04)*

- Can help reduce prescribing

The prompts were perceived by many GPs as being a tool which could lead to an overall reduction in antibiotic prescribing rates. This reduction in prescribing was reported as being an effect which may occur for the individual GP, colleagues, the practice, or all areas involved with the prompts.

*"Oh I would have thought they will have reduced the amount of antibiotics prescribing" (P07)*

- Not needed as guidelines already followed

A key barrier to using the prompts was that some GPs reported not needing them as they claimed that they were already following the advice recommended in the guidelines and had their own methods and procedures for doing this. GPs in this group did not report any problem with the functions or features of the prompts specifically, but simply that they were not needed.

*"I mean I don't find or look at them…because I'm usually relatively comfortable with my respiratory management shall we say, I do very few respiratory referrals etc." (P02)*

#### Perceived positive impact on patients

In general, the prompts were described by GPs as having a positive impact on patients and patient care and were perceived as being acceptable to patients. No negative effects or views of the prompts in relation to patients were reported by GPs within this theme.

- Assistance in persuading patients

The prompts were often reported as providing GPs with assistance in persuading patients of the benefits in following the advice recommended in the guidelines. This particularly referred to persuading patients that antibiotics were not necessary for a RTI in patients who the GP perceived as being unwilling to or apprehensive about accepting this advice.

*"There’s always that kind of feeling like 'oh' (they want antibiotics), but actually it’s very good because it's helpful in guiding patients" (P10)*

- Acceptable to patients

The prompts were reported as being acceptable to patients. This was discussed in relation to the various functions and features of the prompts and the way in which they appeared. GPs felt that the information contained in the prompts was useful to patients in relating to a prescribing decision and they could not think of any problems that their patients would have with the system appearing.

*"I think they give confidence to the patient" (P08)*

- Patient information sheet very useful feature

Overall, the patient information sheet was discussed as being one of the most useful aspects of the prompts. GPs felt that the sheets were a very useful feature which could easily be given to patients who had not been prescribed antibiotics or who were receiving a delayed prescription. GPs often mentioned being familiar with the use of patient information sheets and feeling comfortable in using them.

*"It’s quite nice to give an information sheet because it's a sort of reminder for the patient about what we talked about…..it's quite a nice way to reinforce what our conversation has been about" (P06)*

### Usability issues

A number of issues relating to the usability of the prompts were discussed. This included discussion surrounding controlling the prompts, access and content issues, in addition to suggested improvements for the future.

- Easy to control

The prompts were reported as being easy to control when they appeared on the screen. GPs felt that the prompts could easily be controlled as they did not obstruct the computer screen when they appeared during a consultation, and could easily be either viewed or exited with a minimal number of clicks on the screen.

*"I didn't find them particularly intrusive or anything like that, that I didn't want to use them, it was easy to ignore them" (P07)*

- Easy to use

The prompts were perceived as being easy to use both during a consultation or in the GPs own time. GPs reported that the prompts were simple to navigate, it was clear which features were available, and that the content of the prompts was easy to read.

*"It's very easy and simple to click that" (P06)*

- Limited time to read and use

Some GPs reported that the prompts were often not used or used rarely due to the fact that they had limited time during a consultation or within their busy day to read the prompts. This limited time meant that GPs were not able to read and look through all options and features available within the prompts, or select which functions of pages they may wish to use. In some instances, despite being curious about information within the prompts, GPs felt they did not have enough time to consider them, and therefore did not use them.

*"So it was sort of a nice idea but it's just that sort of real pressure on time, thinking you know, I can't go through all of this and it put me off" (P06)*

- Only English language available

GPs in some areas reported that the prompts were difficult to use with a significant percentage of their patients who did not speak or read English. As the prompts were only available in the English language, information sheets could not be printed and screens could not be displayed to these patients. Some GPs reported that they would have liked to use the prompts if they were available in additional languages.

*"If it’s just in English it's not going to be useful specifically for us…um for our patient population Urdu or Mirpuri" (P01)*

- Additional features

A number of GPs reported that the prompts could be improved by adding various additional features and functions to them. These features included adding further advice for the GP and patient in multimedia format (such as videos), links to additional healthcare services, and information relating to which antibiotic to prescribe.

*"It's quite far-fetched but having some kind of recorded message as well,…….or videos detailing about you know…coughs, colds, not needing antibiotics, not needing consultations with the GP as well" (P01)*

- Simplify further

Some GPs felt that the features, functions and content of the prompts could be simplified further. GPs acknowledged that the information was already concise, but felt that further simplifications could be made to make the prompts even easier to read and use in practice. Some suggestions for this included removing the number of options available on the menu page and reducing text.

*"Well I don't like it when you have to go through several sub-menus really…and a lot of them had more buried you know" (P04)*

- Increase visibility of prompts

It was also reported that the prompts would have been easier to use if they had been more visible on the computer screen during a consultation. GPs reported that they would have used the prompts more often if they had noticed them more and felt that other GPs may have done the same. Suggestions for increasing the visibility of prompts included: making them move across the screen, making them flash, or using brighter colours.

*"I think they just didn't attract your attention away from what you were doing to notice them, so if they were somehow made to stand out more, or moved across to a different part of the screen this would help me" (P12)*

#### Description of implementation staff findings-communication difficulties within practices

The key finding in the implementation staff interviews related to communication difficulties within practices. This was primarily due to the fact that staff members were not being made aware of the surgeries’ participation in the study. Two sub-themes were identified within this theme.

- Delays due to practice staff unawareness of studies

Due to the fact that staff members within each practice were often not aware of the study, the implementation of the computer prompts was often delayed. This was due to the fact that permission for the study IT company to implement prompts on the practice system could not be given until a staff member who knew about the study confirmed participation. It was often the case that no other members of the practice staff had been informed of the study, therefore delays in this process were common.

*“we soon learned that they had absolutely no clue or awareness about the study……the study just ends up running for 18 months if not longer and I think a lot of that frankly was us not being able to get hold of the right person” (P IS 2)*

- Improvements to staff awareness needed

All implementation staff reported that in future, measures should be taken to improve practice staff awareness of involvement in the study. This was seen as essential in order to improve the speed and efficiency of implementation procedures.

*“In terms of pressing the practice…’well look- you have signed up for this thing, so can you please make your practice aware, maybe during the next practice meeting’…..and that would have made all the difference actually…so there was just a complete lack of awareness in my opinion” (P IS 2)*

### Questionnaire study

#### Response rates

83 GPs completed a questionnaire (56 control group and 27 from the intervention group). Practices could take part in the questionnaire anonymously; however there was an option to provide the study team with a postcode in order to provide some data on the location of surgeries. In total practices from 54 different postcodes across the country took part in the survey (there were 21 different postcodes in the intervention group and 33 in the control group).

### Outcome expectancy and self efficacy questionnaire findings

#### Item analysis

Cronbach’s alpha for the outcome expectancies questionnaire was 0.73 and for the self-efficacy questionnaire was .84, indicating an acceptable level of internal consistency.

#### Responses to outcome expectancy and self-efficacy questionnaires

Tables 6 and 7 present the responses for each group to all theory based questionnaire items. Overall the strongest trends in the outcome expectancies questionnaire related to most GPs reporting that treating a patient without antibiotics would help in reducing antibiotic resistance and educate patients that antibiotics are not always necessary. Most GPs were also in agreement that non-prescribing would result in a consultation taking longer to complete.

**Table 6:** **Responses to each item of the outcome expectancies questionnaire**

	**Strongly disagree**		**Disagree**		**Neither agree nor disagree**		**Agree**		**Strongly agree**	
**For each option please rate your level of agreement using the scale below to the following statement: “If I treat a patient with an RTI without prescribing antibiotics....”**	**Intervention**	**Control**	**Intervention**	**Control**	**Intervention**	**Control**	**Intervention**	**Control**	**Intervention**	**Control**
I will help reduce antibiotic resistance	0	0	0	1.8	22.2	5.4	51.9	62.5	25.9	30.4
This will help to educate the patient that antibiotics are not always necessary for treating RTI.	0	0	0	0	18.5	0	40.7	51.8	40.7	48.2
The patient will be less satisfied with the outcome of the consultation.	14.8	3.6	18.5	30.4	47.1	57.1	18.5	8.9	0	0
The patient will be at risk of developing further clinical complications.	0	0	35.7	14.8	44.4	39.3	25	37	3.7	0
The consultation will take longer (in order to explain non-prescribing decision to patient).	3.7	0	3.7	14.3	14.8	10.7	66.7	64.3	11.1	10.7
The patient will be more likely to re-consult with the same problem.	3.7	1.8	44.4	39.3	22.2	21.4	29.6	37.5	0	0
The patient is more likely to book an appointment with another GP in future.	0	1.8	51.9	33.9	18.5	42.9	29.6	24.1	0	0

Figures are percents.

N = 83 (27 intervention group, 56 control group).

**Table 7:** **Responses to each item of the self-efficacy questionnaire**

	**Intervention group**											**Control group**										
**How certain are you that you could treat a patient with an RTI without an immediate prescription of antibiotics? Please use the scale below to rate each statement**	**0**	**1**	**2**	**3**	**4**	**5**	**6**	**7**	**8**	**9**	**10**	**0**	**1**	**2**	**3**	**4**	**5**	**6**	**7**	**8**	**9**	**10**
When a patient DOES want antibiotics	11.1	0	0	3.7	0	48.1	0	11.1	7.4	3.7	14.8	1.8	0	18	5.4	8.9	37.5	12.5	17.9	12.5	1.8	0
When a patient DOES NOT want antibiotics	11.1	7.4	0	11.1	0	0	0	11.1	22.2	7.4	29.6	1.8	0	0	0	0	46.4	0	5.4	23.2	23.2	0
When you are in a rush	11.1	0	0	11.1	0	33.3	3.7	14.8	0	3.7	22.2	1.8	1.8	1.8	8.9	8.9	23.2	16.1	26.8	8.9	1.8	0
When you have plenty of time to talk to a patient	11.1	0	0	0	0	18.5	3.7	11.1	11.1	18.5	25.9	1.8	0	0	1.8	0	19.6	12.5	16.1	33.9	14.3	0
When you think a patient is at LOW risk of developing further medical complications	11.1	0	0	0	0	29.6	0	0	11.1	18.5	29.6	1.8	0	0	1.8	0	19.6	5.4	14.3	25.7	21.4	0
When you think a patient may be at HIGH risk of developing further medical complications	11.1	0	22.2	11.1	11.1	14.8	3.7	7.4	0	18.5	0	7.4	10.7	44.6	17.9	0	5.4	7.1	3.6	3.6	0	0
When a patient has used self-management techniques for the RTI before consulting	11.1	0	0	14.8	0	33.3	11	0	7.4	18.5	3.7	1.8	3.6	19.9	10.7	14.3	23.2	10.7	8.9	5.4	3.6	0

Figures are percents.

N = 83 (27 intervention group, 56 control group).

In the self-efficacy questionnaire, GPs generally reported that they are more confident in treating a patient without antibiotics: when a patient does not want an antibiotic prescription, when a patient is at low risk of developing further complications and when there is plenty of time to talk to the patient.

#### Group differences across theory measures

The Kruskal-Wallis analysis of variance test was conducted on the data (as recommended by Field 2009, due to preliminary tests indicating that data was not normally distributed [26]). There was a significant difference between the intervention and control group in relation to the self-efficacy score (H = 5.69, p = 0.02). The results indicated that the intervention group reported a significantly higher self-efficacy score than the control group. No significant difference was observed in the outcome expectancies score between the two groups (H = 0.58, p = 0.45).

### Intervention evaluation questionnaire

Tables 8 and 9 present all responses to the intervention evaluation questionnaire (completed by GPs in the intervention group only).

**Table 8:** **Responses to sections 1-3 of intervention evaluation questionnaire**

	**Agree strongly**	**Agree**	**Disagree**	**Disagree strongly**
**Software**				
The prompts were easy to read	0	51.9	37.0	11.1
There were no problems accessing the prompts	0	59.3	25.9	14.8
The program was too slow	0	18.5	70.4	11.1
The prompts were difficult to access	11.1	18.5	59.3	11.1
The prompts appeared during consultations for patients with RTIs	0	55.6	29.6	14.8
The prompts appeared at an appropriate time during a consultation	0	51.9	22.2	22.2
The prompts appeared at an inappropriate time during a consultation	7.4	18.5	51.9	18.5
**Prompt Type-Useful in practice**				
Sore throat/pharyngitis/tonsillitis	3.7	44.4	37.0	14.8
Cough/acute bronchitis	3.7	44.4	37.0	14.8
Otitis media	3.7	44.4	37.0	14.8
Rhinosinusitis	3.7	37.0	44.4	14.8
Common cold	3.7	44.4	37.0	14.8
**Use During Consultation**				
The prompts are easy to use during a consultation for RTI.	3.7	44.4	37.0	14.8
There are problems using the prompts *during* a consultation for a RTI.	7.4	29.6	55.6	7.4

Figures are percents.

N = 27 (intervention group only).

**Table 9:** **Responses in percentage to section 4.1 of intervention evaluation questionnaire**

	**Agree strongly**	**Agree**	**Disagree**	**Disagree strongly**
**Guidelines**				
I am familiar with the NICE guidelines for antibiotic prescribing in RTI.	3.7	0	14.8	81.5
I agree with the NICE guidelines for antibiotic prescribing in RTI (which recommend limited prescribing of antibiotics for RTIs).	18.5	55.6	22.2	0
**Communication satisfaction**				
I was satisfied with the level of communication within the practice relating to the trial.	3.7	55.6	18.5	22.2
Communication within the practice relating to the trial could have been improved.	3.7	37.0	29.6	29.6

N= 27 (intervention group only). NICE= National Institute for Clinical Excellence. RTIs= respiratory tract infections.

#### Software

Most GPs agreed that the prompts were easy to read and access, and that the speed of the program was not too slow (for example 80% of GPs agreed that the program was not too slow, and 51% of GPs felt that the prompts appeared at an appropriate time during a consultation). Most GPs felt that the prompts appeared appropriately during consultations for RTI, however approximately 25% of GPs reported that the prompts appeared inappropriately during a consultation.

#### Prompt type

There was little difference in GPs’ views of how useful prompts were in supporting practice across the different RTI conditions. GPs were divided approximately equally in their views as to whether or not the prompts were useful in supporting practice for these conditions (48% vs 52%).

#### Use during consultation

Views were also divided as to how easy the prompts were to use during a consultation. Approximately 48% felt the prompts were easy to use, and 63% felt that there were no problems using the prompts during a consultation.

#### Guidelines

Around 95% of GPs reported that they were not familiar with the NICE guidelines for antibiotic prescribing in RTI, however approximately 75% reported that they did agree with the guidelines.

#### Communication

Most GPs were satisfied with the level of practice communication during the trial and did not think that communication could have been improved (approx. 60%). However, around 51.9% of GPs reported that they did not discuss the trial with other members of staff before it began and 70.4% did not meet with colleagues to discuss the prompts during the trial.

## Discussion

The trial adopted a pragmatic approach in which the intervention was introduced into practice systems remotely, in the same way in which it might be rolled-out into routine practice. The evidence from this process evaluation suggests that GPs’ awareness of the implementation of the system into their practice may be a key factor influencing the utilisation of the intervention. Specifically interviews revealed that GPs who were aware of the implementation of the prompts reported understanding their purpose and feeling confident in using them if they chose to. However, GPs who had not been made aware that the prompts were going to be appearing, reported feeling confused when they saw the prompts appear on the screen and did not fully understand what they were for or how they could be used. This finding would imply that GPs who worked in practices which informed staff that the prompts would be appearing on their screens and provided details of this may be more likely to use and engage with the prompts. This theme was also reflected in the implementation staff interviews, in that one of the key findings related to communication difficulties within practices. Implementation staff revealed that a lack of internal communication across practice staff resulted in severe delays in the prompts being implemented onto some practice systems. In addition, the questionnaire study indicated that approximately half of the GPs reported that they had not discussed the trial with colleagues before it began and around 70% did not discuss the prompts with colleagues during the trial.

This finding is consistent with a previous evaluation of an intervention for RTI, which reported that 67% of GPs had not discussed the trial within the practice before it began [27]. In this instance, poor communication was directly linked to guideline non-adherence. In addition, systematic reviews of interventions delivered to healthcare professionals have also identified poor communication as a major influence on trial outcomes [28-30]. Furthermore, although the sample of implementation staff used in this study was relatively small, the findings are also reflected in the evaluation of similar trials investigating GP interventions. For example, practice staff discontent and communication difficulties, delays in implementation and surgeries requiring several reminders in order to install intervention software have also been previously reported [27,31,32]. In one case reported, the authors concluded that internal communication problems had resulted in making the trial difficult to run and implement in a total of 25% of all practices involved [27]. This finding is particularly interesting as a number of measures were put in place during the trial to ensure dissemination of trial information within each practice. This included information sheets and a link to a demonstration video which were sent directly to practice managers and senior GP partners asking for the dissemination all GPs within the surgery. The finding therefore also suggests that an intensive and recordable form of raising awareness of an intervention is required to ensure that all members of staff within a practice are contacted. The findings also suggest that further research is required to identify the most efficient and acceptable ways of disseminating trial information within a GP practice. Evidence suggests that trial information is often not disseminated within a practice and can have an impact on trial success. Furthermore, evidence from this evaluation suggests that measures used to increase awareness were not effective. It is possible that a number of factors may influence the adoption and dissemination of an intervention, including issues such as competing time demands, staff availability and differing organisational structures across surgeries. Therefore, an identification of effective dissemination techniques could greatly benefit the efficiency of future implementation trials.

The findings of the GP interviews also appear to be consistent with self-determination theory [22]. When discussing the use of prompts, GPs reported finding the prompts useful if the information supported their own prescribing decision. This suggests that rather than changing behaviour or persuading them not to prescribe antibiotics, the GPs thought the prompts were useful if they were perceived as supporting a decision to adhere to the guidelines which the GP had made autonomously. This is consistent with self-determination theory which suggests that techniques which encourage autonomy would result in greater compliance and engagement. In addition, GPs also reported finding the prompts useful as they were easy to control. This would also be consistent with self-determination theory as placing the GP in control of the prompts and not forcing information to appear increases autonomy and according to the theory would make an individual more likely to engage in a behaviour. Furthermore, this finding was also supported in the prompts development study [16] as GPs reported that if they felt that they had control to choose to use the prompt and that it was supporting them, they would be likely to use it. This repeated finding therefore suggests that GPs may be more likely to engage in a computer-delivered intervention if it is perceived as being easy to control and not enforcing recommendations but supporting GPs autonomy.

The intervention evaluation questionnaire revealed that, overall, software and usability of the prompts were rated highly by GPs (however as intervention fidelity was only assessed by voluntary self-report data, it is possible this may not have been experienced by all GPs within the trial). Half of the GPs reported that the prompts were useful in supporting practice, however half reported that they were not useful. From these figures it is hard to assess GPs’ views of the prompts, however findings from the qualitative evaluation can provide some insight into this issue. The interview study revealed that some GPs viewed the prompts as unnecessary, as they felt that guidelines were already being followed, whereas others viewed the prompts as useful reference tools which could support their prescribing decision. Therefore it is possible that GPs who rated the prompts as not being useful in supporting practice may have done this as they view the prompts as unnecessary due to guidelines already being followed.

In the questionnaire study, the intervention group reported significantly higher levels of self-efficacy in relation to managing a patient who was at high risk of developing complications without using antibiotics. This finding is also supported by those of the qualitative interviews, in that GPs reported that the prompts were useful in acting as a reference tool (for information relating to the NICE guidelines in RTI), a tool which could support their decision, and a tool which could help in persuading patients to accept advice. However, these findings should be interpreted with caution, as analyses of individual items found a significant difference on only one individual questionnaire item, relating to a single aspect of self-efficacy (managing a patient who is at a high risk of developing complications). Future research could extend the variation of questions relating to patients who are at high risk of complications to confirm the possibility of this finding.

### Limitations

The voluntary nature of participation in the study may have led to an unrepresentative sample of GPs taking part. In particular, GPs with an interest in research or antibiotic prescribing in RTI may have been more likely to participate, which may have led to responses which were more in favour of guideline adherence or use of prompts. Some negative views were expressed towards the prompts, however in general GPs viewed the prompts in a positive light (which may indicate a research bias sample who are have an interest in interventions of this nature). GP practices participating in the study were members of the CPRD (Clinical Practice Research Datalink), had voluntarily agreed to take part in the trial, and had then voluntarily agreed to participate in the process evaluation study which may have resulted in more research experienced practices expressing their views. It is likely that practices who are not experienced in research may have held different opinions towards the intervention and implementation.

The sample may also have been biased towards GPs who were aware of the study, and not represented the views of GPs who were unaware of the trial or prompts. GPs in this group may have been less likely to complete a questionnaire if they were unsure what the prompts were and as a result a sample which over-represented the views of GPs aware of the trial may have occurred. Furthermore, the voluntary nature of participating in the evaluation study also prevented an overall figure being obtained of the number of GPs who were not aware of the intervention and therefore can only provide an indication. In this instance, making the questionnaire and interview a compulsory part of GP participation in the trial would assist in ensuring that views which represent all GPs who took part in the study are presented in future research.

In addition, as the evaluation related to a cluster randomised trial, demographics were collected at practice level and did not relate to individual GPs. In future collecting individual GP data such as age and experience may provide additional information which can be used to gain a deeper understanding of GPs views and opinions towards an intervention.

As the study aimed to evaluate intervention use and implementation, the sample consisted only of practitioners and staff within the trial. In future, gaining suitable permissions to identify and invite patients for an interview may identify additional issues relating to the way in which GPs use an intervention in practice. Patient interviews could identify issues relating to patient satisfaction and willingness to adhere to advice provided during the intervention.

Furthermore, the evaluation of the behaviour change theory and techniques used in the intervention could have been enhanced if a theoretical mapping process had occurred prior to the intervention development. In the trial, once theoretical constructs had been selected from the literature, development of the intervention began. However, the development of the intervention content at the point of theory selection could have been mapped onto related theoretical domains identified by Michie et al. [33] for use in behaviour change interventions. A benefit of following this process is that once complete, the theoretical domains can be mapped onto corresponding evidence based behaviour change techniques, which research has identified as being beneficial in relation to the specific domains identified [33,34]. Once the evidence based behaviour change techniques have been selected, these can then be operationalized into a suitable format for the intervention. This technique would have allowed a closer evaluation of the theoretical constructs and specific behaviour change techniques used within the content of the intervention. The evaluation of future interventions could be improved if a more empirical theoretical mapping approach is used in the development of such systems. This technique creates a more evidence based method and transparent process which can benefit both concise evaluation and future research.

## Conclusions

A remotely-installed, computer-delivered intervention for use at the point-of-care is an acceptable tool for use in general practice. However, problems with internal communication within practices resulted in difficulties implementing the intervention and may have reduced GP engagement with the system. The findings suggest that the utilisation of future interventions could be increased if measures are included to ensure that practice staff and GPs are made aware of the implementation of an intervention prior to it commencing. However, care should be taken to ensure that any promotion or information relating to a point of care system is as minimalistic as possible and does not in itself provide a form of intervention which could influence GP practice.
